# Field emission in vacuum resonant tunneling heterostructures with high current densities

**DOI:** 10.1038/s41598-023-44900-2

**Published:** 2023-11-08

**Authors:** Michael V. Davidovich, Igor S. Nefedov, Olga E. Glukhova, Michael M. Slepchenkov, J. Miguel Rubi

**Affiliations:** 1 Department of Physics, Saratov State University, Astrakhanskaya street 83, Saratov, Russian Federation 410012; 2 RUDN University, 6 Miklukho-Maklaya St, Moscow, Russian Federation 117198; 3 Laboratory of Biomedical Nanotechnology, I.M. Sechenov First Moscow State Medical University, Bolshaya Pirogovskaya Street 2-4, Moscow, Russian Federation 119991; 4 Department of Condensed Matter Physics, University of Barcelona, Marti i Franquès 1, 08028 Barcelona, Spain

**Keywords:** Applied physics, Electronics, photonics and device physics

## Abstract

We analyse the steady-state thermal regime of a one-dimensional triode resonant tunnelling structure. The high currents generated by resonant tunnelling produce a large amount of heat that could damage the structure. Establishing the conditions under which it can operate at optimum efficiency is therefore a problem of great relevance for applications. The tunnel current is found via eigenvalues of the Schrödinger equation in quantum wells. By calculating the current generated in the device and using the energy conservation law in the electrodes, the temperature reached is obtained for different types of electrodes and the importance of heat conduction and thermal radiation is analysed. In the cases discussed, conduction is dominant. When the electrode material is copper, the temperature reached is similar to that of the thermostat for a wide range of electrode lengths, whereas when the cathode material is diamond-graphite and the anode material is copper, the temperature increases significantly as a function of length. The results obtained allow the temperature to be controlled for optimum performance of the field-emitting triode structures.

## Introduction

The tunnel effect is of utmost importance in electronics as it empowers electrons to cross potential barriers. A considerable surge in electron current occurs when electrons go through a double-barrier structure at an energy level close to that of one of the metastable levels of the quantum well. This phenomenon is known as the resonant tunneling effect (RT), which was initially studied by Esaki in semiconductor structures^1^ . It makes it possible to increase the field emission current density by several orders of magnitude, which is important for example in vacuum current sources. The high current generation produces a large amount of heat that could damage structures. An important question is to establish the conditions under which high currents can be generated without damaging the structure due to high temperatures that those currents could cause.

Typically, semiconductor resonant tunneling diodes (RTDs), resonant tunneling transistors (RTTs), and cascaded, tilt-shunt lasers are fabricated with quantum heterostructures (e.g., of the AlAs/GaAs type)^2–15^. RT has also been considered in carbon nanotubes^16^, graphene structures^17^, and diode and transistor structures with quantum dots^18–21^, including single-electron transistors. In solid-state RTDs, the height of barriers is controlled by the drain voltage (anode). In field-emitting vacuum structures, it is possible to create additional barriers by introducing grating electrodes, and potentials with one, two or more quantum wells. The use of several grids with the same grid potential $U_g$ is convenient for using RTTs as high-current sources with field emission for vacuum devices, even in the THz range.

**Figure 1:**
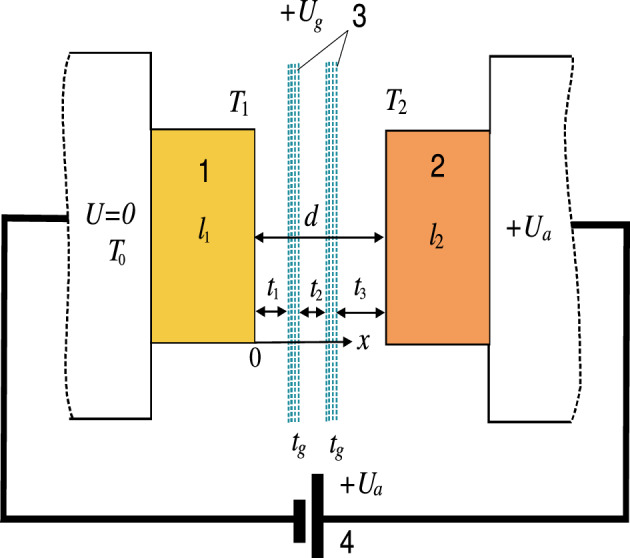
Scheme of a field-emission triode structure.

In the case of quantum superlattices with layer sizes less than 1 nm, tunneling problems can be solved by using quantum transport approaches in combination with the tight binding method and density functional theory^22^. If the sizes of barrier regions and quantum wells of the order of 1 nm or more, it is more expedient to determine the quantum potential of such classical superlattices solving the Schrödinger equation together with the Poisson equation, and then determine the tunneling current, correcting it for the space charge. This is necessary for high currents which can be achieved in structures with several barriers and quantum wells created by several electrodes when electrostatic potentials are applied to them. The simplest structure of this type is a vacuum nano-diode with a grid potential of the order of the anode potential In this case, two barriers appear with a well between them, the depth of which can be controlled by the grid. In the case of a tetrode, it is convenient to combine two grids under one potential, i.e. perform a triode. In this case, two wells of the same depth and three barriers appear. The length of such structures does not exceed 10 nm, This makes it possible to consider the transport as ballistic and not to take into account the scattering of electrons by phonons, i.e. supposing that the electrons move along the entire structure in the quantum potential *V*(*x*) with a complex multiwell profile. This profile can be calculated using the classical method of multiple images with the introduction of experimentally determined work functions into the formulas^23–25^. This makes it possible to accurately determine the quantum potentials and transparency of structures in the one-electron approximation. The field emission current density depends on *V*(*x*) and the electron concentration in the cathode material. However, the limiting current density

1 $$\begin{aligned} j_{\text{lim}}=em_e\pi ^{-2}\hbar ^{-3}E_{F_c}/4 \end{aligned}$$

is not achievable, since it means full transparency at all energies which is not possible due to the the quantum nature of the system. It was shown in^23^ that densities lower by 2-3 orders of magnitude are achievable in RTTs. In the previous formula 1*e* is the electron charge, $m_e$ is its mass, and $E_{F_c}$ is the Fermi energy of the cathode. Further, the subscript $_c$ corresponds to the cathode, $_a$ to the anode, and $_g$ to the grid. Also according to Fig. 1 we will assign index 1 to the cathode, and 2 to the anode. For copper, $E_{F_c}=7$ eV, $j_{\text{lim}}=4\times 10^{15}$ A/m$^2$, and achieving current densities of value $j=10^{12}$ A/m$^2$ requires an appropriate thermal regime. It should be noted that in solid-state RT structures the barriers decrease by a factor of the permittivity $\epsilon , \sim 11 - 14$; therefore, the barrier height does not exceed 0.5 eV at a substantially lower effective carrier mass. This and a small size of the barriers reduce operating voltages which eliminates the electrical breakdown. An increase of the current in metal/dielectric/metal/dielectric/metal nanostructures compared to vacuum structures is possible with the use of appropriate dielectrics. These are crystalline diamond, $\epsilon =5.6$, CVD diamond, amorphous diamonds (diamond-like amorphous carbon)^26^. The high thermal conductivity of diamond allows to equalize the temperatures of the cathode and anode.

**Figure 2:**
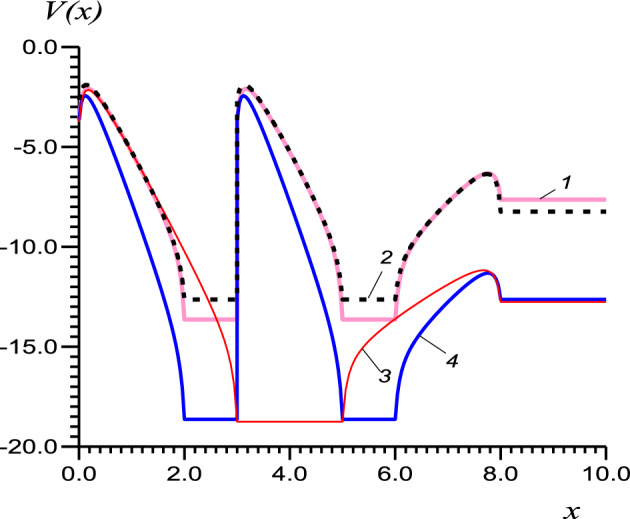
Profiles of the complex barriers (in eV) in two-well (1,2,4), and one-well structures (3) with two grids under equal potentials. The structure parameters are (in eV): grid and anode quantum potentials $V_g=10,\, V_a=5$ (1,2), and $V_g=15,\,V_a=10$ (3,4); $W_c=3,\, W_g=4$ (1,3,5) and $W_c=3.6,\, W_g=3$ (2); $t_1=t_2=t_3=2$ nm, $t_g=1$ nm (1,2,4), and $t_1=t_2=3$ nm, $t_g=2$ nm (3); $W_a=4.5$, and Fermi energy $E_F=7$.

**Figure 3:**
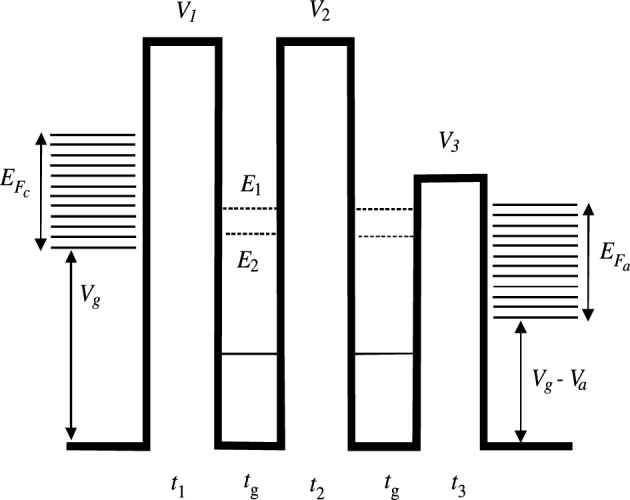
Rectangle approximation of the quantum potential profile. Two meta-stable levels are shown by dashed lines and one stable level by solid lines. $V_a=eU_a$, $V_g=eU_g$.

In this article, we consider vacuum RT triode-type structures recently studied in^23^, see Fig. 1, where the vacuum and grid electrode regions are several nm in size. Such sizes allow the use of low voltages in the order of several volts. Potential profiles *V*(*x*) in which quantum barriers and quantum wells alternate, see Fig. 2, can be calculated by applying the multiple imaging principle^24,27^. We have solved the one-dimensional one-particle Schrödinger equation (SE) in the gaps between the electrodes^23^ and the one-dimensional stationary problem of tunneling and heat transfer in the model double-well three-barrier structure (Fig. 3). In such RTT structures, In addition, it is possible to use the experimentally determined electrode work functions and thus correctly describe the quantum structure without solving the multiparticle SE inside the electrodes. This fact makes it possible to solve the one-dimensional SE in the intervals between the electrodes^23^.

Multiparticle SE is usually approximated by means of the density functional theory (DFT), which allows us to determine the work function of a metal, although with a significant error compared to the experiments^25^. For several electrodes, DFT was used for tunneling in graphene structures for which phenomenological potentials included in the Hamiltonian are known^1,22–31^. In this case, an approach based on nonequilibrium Green-Keldysh functions was used. The distribution of potentials in such structures is more accurate and easier to obtain using the multiple imaging method taking into account the experimental values of the work functions of all electrodes^23,24^ ($W_c,\,W_g,\, W_a$ are the work functions of the cathode, grid, and anode, respectively), and then solving the SE. At high current densities, this distribution should be refined based on the solution of the Poisson equation.

Due to heating, semiconductor heterostructures do not allow high-current electron sources. Vacuum electronics require high current vacuum sources of field emission^32^ , and carbon nanotube cold cathodes, employable in electron guns^33^. Two-well triode resonant tunnel structures are potentially promising^23^. A structure consisting of two layers and three barriers can be obtained by applying the same high positive potential to a double grid with a gap between the electrodes, see Fig. 2, thus forming a double well, as shown in the figure, with the possibility of resonant tunnelling. By considering the anode as a grid, it is possible to accelerate the formed electron beam and use it in microelectronic and nanoelectronic vacuum devices, in particular, in travelling wave tubes in the THz range.

It is therefore very important to study the thermal regime by calculating the temperature that the structures reach as a function of the current. This is the main objective of this article.

To this purpose, we will solve a stationary one-dimensional tunneling and heat transfer problem in a two-layer, three-barrier structure, see Fig. 3. In these RTT structures, it is theoretically possible to obtain current densities one to three orders of magnitude lower than the maximum densities possible in the hypothetical case of a structure transparent to all incident electron energies. With such enormous densities, it is necessary to take into account the heating of the structure, which can be very strong, and also the effect of the charge density on the potential distribution by solving the Poisson equations in a self-consistent way. We will not consider the latter problem here. Our aim is therefore to identify the regimes in which the heating is not so strong as to damage the structures. A rough estimate shows that in such regimes the potential change does not exceed a few percentage points, which is quite acceptable.

The article is organised as follows. In “Statement of the problem” section, we present the resonant tunneling triode structure. In “Calculation of the tunneling current”, “Calculation of the transparency coefficient” and “Energy eigenvalues and tunnel current” sections, we introduce the method used to calculate the tunnel current and give the expression of this current. “Stationary thermal regime of a one-dimensional tunnel structure” section is devoted to analyse the stationary thermal regime and in “Poynting vector and radiative heat transfer” section we investigate the role of radiative heat transfer in the stationary temperatures reached by the electrodes. Finally, in “Conclusions” section we summarize our main conclusions.

## Statement of the problem

The system to be studied is shown in Fig. 1 where $U_{c}$, $U_{g}$, $U_{a}$ represent the electrostatic potentials at the cathode, grid and anode, respectively, whose origins are at the cathode. Quantum potentials measured in eV are denoted by *V*. Their origins are at the bottom of the wells, so the values are positive. Furthermore, we assume that $U_g\ge U_a$, so the depth of the wells is the same, while the maximum quantum potentials at the cathode and anode are equal and given by

2 $$\begin{aligned} \begin{array}{cc} V_c=eU_a+E_{F_c},&V_a=e(U_g-U_a)+E_{F_a}, \end{array} \end{aligned}$$

where $E_{F_c}$ and $E_{F_a}$ are the Fermi energies at the cathode and anode. Due to the small thickness of the grid electrodes, we do not need to consider the energy levels associated with their atomic structure and located below the bottom of the wells, i.e. we do not consider the tunnelling processes associated with the capture of electrons at these levels and their re-emission, as well as the scattering processes in the grids. This is allowed if $U_g\ge U_a$ since all levels below the bottom of the wells cannot be metastable. In reality, the mean free path of electrons in the structures corresponding to the grids is more than an order of magnitude larger than their sizes. Metastable (complex) levels are possible in the region of the well. Tunneling for energies equal to the real part of the energies of these levels occurs with little or no reflection, i.e. these levels are suitable for RT. In the case $V_g-V_a>E_{F_a}$, the formation of stable levels is also possible. These deep levels do not participate in tunnelling, however, a large potential in the grid leads to a strong decrease of the height of the barriers $V_1$ and $V_2$, and therefore to a strong increase of the current.

All potentials in Fig. 2 are determined by the work function of the electrode and the width of the vacuum gap. The transparency of the structure quantified by *D*(*E*) is usually exponentially small between the levels, so integration over the whole energy domain can be replaced by summation over the levels, assuming that electrons move to these levels from their close vicinity, and from them to the anode. The availability of several or at least one of these levels above the lower part of the anode conduction band is necessary for RT. We will approximate the real potentials *V*(*x*) of Fig. 2 by three rectangles (see Fig. 3) and solve the SE by the method of transfer matrices^23–25^, using only five matrices. For a more accurate solution of the tunneling problem with the actual profile of Fig 2, we should use several hundred transfer matrices corresponding to the divisions of the interval between the cathode and the anode^23^. The use of only five of them gives us an acceptable accuracy of the current that allows us to properly estimate the stationary temperature of the structure, which is the objective of this work.

## Calculation of the tunneling current

We consider the resonant tunneling from the level with energy $E_1$ and width $\Delta E_1$ in which each electron releases an amount of energy $E_{F_c}-E_1$ to the cathode, and assume that all the electrons in the specified band tunnel and the remaining electrons are reflected. We will consider that the specified band $\Delta E_1$ is such that $|E-E_1|\ge \Delta E_1$. The current density for a transparency coefficient $D_1$ in the band area and $D_1=0$ outside is

3 $$\begin{aligned} j=\frac{em_eE_{F_1}D_1\Delta E_1}{2\pi ^2\hbar ^3}\left( 1-\frac{E_1}{E_{F_c}}\right) \end{aligned}$$

measured in A/m$^{2}$, where $m_e$ is the electron mass. This expression is obtained from the electron velocity *v* distribution at zero temperature giving

4 $$\begin{aligned} dn_{v_x}=\frac{m_e^2}{4\pi ^2\hbar ^3}v_x(v_F^2-v_x^2)dv_x =\frac{m_e}{2\pi ^2\hbar ^3}(E_{F_c}-E)dE. \end{aligned}$$

where the Fermi energy is

5 $$\begin{aligned} E_{F_c}=\frac{(3\pi ^2)^{2/3}}{2m_e}\hbar ^2n^{2/3}, \end{aligned}$$

and $v_F$ is the Fermi velocity. The number of electrons $n_0$ with momenta $p_x,p_y,p_z$ having energies smaller or equal to $E=(p_x^2+p_y^2+p_z^2)/(2m_e)$ is given by

6 $$\begin{aligned} n_0=\frac{8\pi }{3(2\pi \hbar )^3}(p_x^2+p_y^2+p_z^2)^{3/2}= \frac{8\pi (2m_eE)^{3/2}}{3(2\pi \hbar )^3}. \end{aligned}$$

The number of incoming electrons at the barrier with momentum $p_x$ and velocity $v_x$ is $dn_{v_x}$, see Eq. 4. Using the relation $dv_x=dE/\sqrt{2Em_e}$ and the expression for the current density

7 $$\begin{aligned} j=e\int _0^{v_x=\sqrt{2Em_e}}D_{v_x}dn_{v_x}, \end{aligned}$$

we obtain

8 $$\begin{aligned} j=\frac{em_e}{2\pi ^2\hbar ^3}\int _0^{E_{F_c}}\,D(E)(E_{F_c}-E)dE \end{aligned}$$

where *D*(*E*) is the energy-dependent transparency coefficient to be calculated in the next section. The current density can be approximated by the formula

9 $$\begin{aligned} j=\frac{em_e}{2\pi ^2\hbar ^3}\sum _{n=1}^N\,D_n\Delta E_n(E_{F_c}-E_n). \end{aligned}$$

## Calculation of the transparency coefficient

To find the tunneling current, we first calculate the transmission coefficient for electrons moving from the cathode to the anode by solving the SE using the transfer matrix method for the potential shown in Fig. 3. Details of the quantum potential profile calculations can be found in^23^. The form of *V*(*x*) is approximated by a step-wise function with a transfer matrix for each step. The *n*-th step transfer matrix ${\mathbb {A}}^n$ has elements

10 $$\begin{aligned} \begin{array}{ccc} a^n_{11}=a^n_{22}=\cos {(k_nt_n)},&a^n_{12}=-ik_n^{-1}\sin {(k_nt_n)},&a^n_{21}=-ik_n\sin {(k_nt_n)}, \end{array} \end{aligned}$$

where $k_n=\sqrt{\mu (E-V_n)}/\hbar$ for the region above the barrier, and $k_n=i\sqrt{\mu (V_n-E)}/\hbar$ for the region below the barrier. Since in the wells $V=0$, the elements of the transfer matrix ${\mathbb {A}}^g$ for wells or grids are

11 $$\begin{aligned} \begin{array}{ccc} a^g_{11}=a^g_{22}=\cos {(kt_g)},&a^g_{12}=-ik^{-1}\sin {(kt_g)},&a^g_{21}=-ik\sin {(kt_g)}, \end{array} \end{aligned}$$

where $k=\sqrt{\mu E}/\hbar$. In Fig. 2, zero energy corresponds to the free electron case, thus $V<0$. Here and in Fig. 3, the energy is counted from the bottom of the well, therefore it is always positive. With this choice, the potential energy of the electrons at the cathode is increased by $eU_g$, and that of those at the anode by $e(U_g-U_a)$. The wave vectors at the cathode and anode are given by $k_c=\sqrt{\mu (E-eU_g)}/\hbar$, and $k_a=i\sqrt{\mu (E-e(U_g-U_a))}/\hbar$, respectively.

The transfer matrix of the whole structure ${\mathbb {M}}$ is then given by ${\mathbb {M}}={\mathbb {A}}^1 {\mathbb {A}}^g {\mathbb {A}}^2 {\mathbb {A}}^g {\mathbb {A}}^3$. The origin of the energies can be placed at any level, for example at the bottom of the cathodic conduction band. In this case, the values entered will change but not the final result. According to Fig. 3, the maximum kinetic energy of electrons incident on the structure is $E_{F_1}=V_g$. The energy of the electrons leaving the anode is equal to $E_{F_1}+V_g-eU_a$. Consequently, we must modify the Eq. 8 obtained when counting the energy from the cathode, taking the lower limit $V_g$ and the upper limit $E_{F_1}+V_g$. At the same time, when calculating *D*, we must consider energies in the domain $V_g<E<E_{F_1}+V_g$.

The wave function at the cathode is $\Psi _c=\exp {(ik_cx)}+R\exp {(-ik_cx)}$, whereas at the anode for the electrons going into the power source is $\Psi _a=\tau \exp {[ik_a(x-d)]}$, where *R* and $\tau$ are the reflection and transmission coefficients. Electrons arriving at the anode have wave function $\exp {[ik_c(x-d)]}$, and the change from $k_c$ to $k_a$ occurs in the anode in the free path length. It should be noted that tunneling to the last turning point occurs without energy loss. Then electrons move quasi-classically in a small path and are accelerated by the anode voltage in the decreasing section of the potential function, see Fig. 2. The energy acquired by the electrons in a free path is released at the anode. Usually, in tunnelling the anode region is not taken into account in a quasi-classical approximation, and the anode wave function is taken at the turning point $d'<d$ and is of the form $\Psi _a=\tau '\exp {[ik_c(x-d')]}$^34^, where $\tau '$ is the transmission coefficient for the case when the anode area is ignored and the wave function at the anode has the form $\Psi _a=\tau '\exp {(ik_c(d-d'))}$. However, the area behind the turning point $d'<d$ contributes to the transmission and has been taken into account in our approach, while at the anode the wave function has the form $\Psi _a=\tau \exp {(ik_c(d-d'))}$.

The quasi-classical method for determining the transparency coefficient, or the method of slowly varying amplitudes, is based on calculating the integral of $\sqrt{\mu (V(x)-E)}$ from the beginning of the barrier to the turning point $d'$ and gives rise to the relation $|R|^2+|\tau '|^2=1$. A more precise calculation of the transmission coefficient $\tau$ by applying the transfer matrix method and taking into account the area beyond the turning point gives the relation $|R|^2+k_a/k_c|\tau |^2=1$. We will call the value $D=k_a/k_c|\tau |^2$ transparency as opposed to transmission $\tau$.

The matrix ${\mathbb {M}}$ accounts for the scattering process of electrons by a quantum potential, connecting the wave function $\Psi _c$ and its derivative on the left-hand side of the structure (at $x=0$) with the wave function $\Psi _a$ and its derivative on the right (at $x=d$) in the form

12 $$\begin{aligned} \begin{array}{l} 1+R(E)=[M_{11}(E)+ik_aM_{12}(E)]\tau (E), \\ ik_c[1-R(E)]=[M_{21}(E)+ik_aM_{22}(E)]\tau (E). \end{array} \end{aligned}$$

From Eqs. 12 it follows

13 $$\begin{aligned} &  \tau =\frac{2}{M_{11}(E)+k_a/k_cM_{22}+ik_aM_{12}-i/k_cM_{21}} \end{aligned}$$

14 $$\begin{aligned} &  \frac{1+R(E)}{1-R(E)}=ik_c\frac{M_{11}(E)+ik_aM_{12}}{M_{12}(E)+ik_aM_{22}} \end{aligned}$$

For the transmission coefficient $\tau '$ at the turning point (assuming $k_a=k_c$) we obtain

15 $$\begin{aligned} \tau '(E)=\frac{2}{M_{11}(E)+M_{22}+ik_cM_{12}-i/k_cM_{21}} \end{aligned}$$

Tunneling is the process of reflection and transmission of electrons coming from the cathode with probabilities $|R|^2$ and $|\tau |^2$, respectively. The probability flux on the left and on the right is continuous and in the absence of reflections at the cathode is $\tilde{j_c}=k_c\hbar /m_e$, and at the anode is $\tilde{j_a}=k_a\hbar |\tau |^2/m_e$^33^. From the continuity of this quantity, if $|R|=0$ it follows that $|\tau |^2=k_c/k_a<1$. Therefore, the transparency coefficient is $D(E)=|\tau |^2k_a/k_c$.

The current density is $j=-e{\tilde{j}}_a$ (due to the negative charge of the electron). The normalization of the wave function used is made with respect to the case of one particle at a unit length per second in the electron flow cross-section. A different normalization was used in formula 6 which corresponds to the actual number of electrons falling on the cathode boundary. The use of the expression $D(E)=|\tau '|^2$ leads to an error in the determination of the coefficient since the motion of the electrons near the anode can be fast in the resonant case. The diagonal elements of the matrix are dimensionless whereas the non-diagonal ones have dimensions of length (for subscripts 12) and inverse length (for subscripts 21).

**Figure 4:**
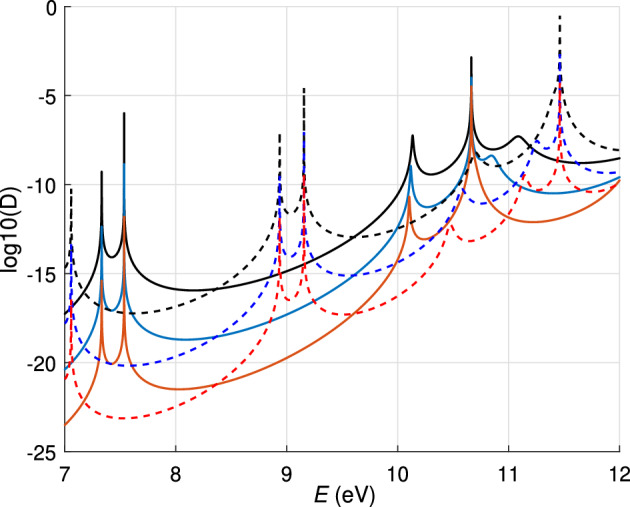
Tunneling coefficient *D* in a logarithmic scale for the two-well structure with parameters: $U_a = U_g = 5$ V. $V_1 =V_2=14$ eV, $V_3=10$ eV, $E_{F_c}=E_{F_a}=7$ eV, $t_1=t_2=t_3= t$. Solid and dashed lines correspond to the structures with $t_g=1$ nm, and $t_g=1.5$ nm, respectively. Black, blue, and red lines show *D* for the width of the barrier $t=0.6$, 0.7, and 0.8 nm, respectively.

Figure 4 depicts the transparency coefficient *D* versus energy *E* for six structures with different widths of the barriers and quantum wells. Since the transparency coefficient is very small outside the resonant peaks, it is plotted in a logarithmic scale. The peaks are very sharp, their heights are given in Tables 1 and 2.

**Table 1:** Energy levels in two-well structure at $U_a=U_g=5$ V.

$E_n$	*t* = 0.6 nm	$t_g$ = 1 nm	*t* = 0.7 nm,	$t_g$ = 1 nm	*t* = 0.8 nm	$t_g$ = 1 nm
$$E_1$$	$$7.3324-1.234\times 10^{-5}i$$,	$$D_1=1$$	$$7.332-2.32\times 10^{-6}i$$,	$$D_1=1$$	$$7.332-4.345\times 10^{-7}i$$,	$$ D_1=1$$
$$E_2$$	$$7.535-4.012\times 10^{-8}i$$,	$$D_2=1$$	$$7.535-2.961\times 10^{-9}i$$,	$$D_2=1$$	$$7.535-2.2\times 10^{-10}i$$,	$$ D_2=1$$
$$E_3$$	$$10.134-0.0062i$$,	$$D_3=2.\times 10^{-8}$$	$$ 10.117-0.005i$$,	$$D_3=2\times 10^{-8}$$	$$8.936-2.16\times 10^{-4}i$$,	$$ D_3=2\times 10^{-9}$$
$E_4$	$10.66-7.55\times 10^{-7}i$,	$D_4=4\times 10^{-5}$	$10.66-4.44\times 10^{-7}i$,	$D_4=4\times 10^{-7}$	$10.66-2.2\times 10^{-6}i$,	$D_4=9\times 10^{-7}$
$J\text{tot}$	$J_1=5.32\times 10^9$		$J_2=1.3\times 10^9$		$J_3=1.86\times 10^8$	

*V* = 14 eV, ${\tilde{V}}$ = 10 eV $E_{F_c}$ = 7 eV, $t_1=t_2=t_3=t$.

**Table 2:** Energy levels in two-well structure at $U_a=U_g=5$ V.

$E_n$	*t* = 0.6 nm	$t_g$ = 1.5 nm	*t* = 0.7 nm	$t_g$ = 1.5 nm	*t* = 0.8 nm	$t_g$ = 1.5 nm
$$E_1$$	$$7.05-1.717\times 10^{-8}i$$,	$$D_1=1$$	$$7.05-1.153\times 10^{-9}i$$,	$$D_1=0.54$$	$$7.05-7.75\times 10^{-11}i$$,	$$ D_1=1$$
$$E_2$$	$$8.93-1.664\times 10^{-4}i$$,	$$D_2=1$$	$$8.93-5.77\times 10^{-5}i$$,	$$D_2=1$$	$$8.93 -2.004\times 10^{-5}i$$,	$$ D_2=1$$
$$E_3$$	$$9.15-1.356\times 10^{-7}i$$,	$$D_3=1$$	$$9.15-1.38\times 10^{-8}i$$,	$$D_3=1$$	$$9.15-1.44\times 10^{-8}i$$,	$$ D_3=1$$
$E_4$	$11.46-9.89\times 10^{-5}i$,	$D_4=2\times 10^{-4}$	$11.46-2.81\times 10^{-6}i$,	$D_4=3.2\times 10^{-5}$	$11.46-2.4\times 10^{-7}i$,	$D_4=5.5\times 10^{-6}$
$J_{\text{tot}}$	$J_4=5.1\times 10^{10}$		$J_5=1.85\times 10^{10}$		$J_6=1.5\times 10^9$	

*V*=14 eV, ${\tilde{V}}$=10 eV $E_{F_c}$=7 eV, $t_1=t_2=t_3=t$.

## Energy eigenvalues and tunnel current

Let us consider the condition for the formation of resonant levels which is an eigenvalue problem. RT corresponds to a zero reflection coefficient $R(E)=0$^6^ from which the characteristic equation follows

16 $$\begin{aligned} -ik_c=\frac{M_{21}(E)+ik_aM_{22}(E)}{M_{11}(E)+ik_aM_{12}(E)}. \end{aligned}$$

In this equation, we have changed the sign of $k_c$ since the wave function of an electron leaving the energy level *E* in the well towards the cathode must be $\Psi _c(x)=\exp {(-ik_cx)}$. However, if $E<V_g$, there are no levels at the cathode into which an electron can escape, therefore, $\Psi _c(x)=\exp {(|k_c|x)}$, i.e. the function decays in the region $x<0$, and electrons can only go to the anode. This means that $k_c=i|k_c|$. Similarly, if $E<V_g-eU_a$, electrons cannot go to the anode. In this case, only a stable level can be formed. Finding metastable levels is important because their location affects the current value and determines the RT current, which is relevant for the design of structures operating under optimal conditions. To pinpoint such levels, we must find all the complex roots of Eq. 16.

To solve Eq. 16 numerically it is convenient to first find the real roots and use them as a starting point for finding the complex roots. The complex energy eigenvalues of the metastable levels $E_n=E_n'-iE_n''$ for six structures are given in Tables 1 and 2. For the first three structures, the width of the quantum well $t_g$ is taken to be 1 nm, and for the rest of the structures $t_g$ = 1.5 nm. The tables also show the values of the transparency coefficients *D* of these structures calculated with high accuracy in the vicinity of the transmission peaks (see Fig. 4). The maximum transmission values given in the peaks of Fig. 4 correspond to the real parts of the energies in the tables. We have considered only the energy levels in the range 7–12 eV since the lower local maxima of the transparency do not show a complete resonant tunneling and their contribution to the current is very small.

As expected, increasing the width of the barriers leads to a decrease in the tunnel current. For structures 1–3, the two lower levels are responsible for the total transmission, i.e. $D=1$. The last high-energy peak has a lower height in all six structures. The increase of the quantum well width from 1to 1.5 nm leads to the appearance of an additional energy level within the considered interval. The corresponding transmission peak is located near the lower edge of this interval and reaches unity. Therefore, the transmission in structures 4–6 has a higher peak at $D=1$ than in structures 1–3. This explains why the total tunnel currents in 4–6 are higher than in 1–3.

The values taken by *D* between maxima are much smaller than $\Delta E_n=E_n''$, which makes it easier to find the complex eigenvalues of the energy. On the other hand, the solution to such a problem allows to optimise the localisation of the levels, which is important for the creation of structures with a suitable thermal regime. Note that we have neglected the backward tunneling effect from anode to cathode, which is justified if the anode voltage is of the order of several volts or more, since the backward current is exponentially small. Backward currents must be taken into account in devices operating at low voltages.

For the calculation of transmission and reflection coefficients, a different approach based on the impedance transform could also be used^6^.

## Stationary thermal regime of a one-dimensional tunnel structure

We consider the stationary state of the RTT structure in which $T_1$ and $T_2$ are the temperatures of the cathode and the anode, and $T_0$ is the temperature of the thermostat in contact with the cathode, the anode, and the power supply. The cathode and anode thermal conductivity coefficients are $k_1$ and $k_2$ and their respective lengths are $l_1$ and $l_2$. The electron mean free path $l_0$ is assumed to be the same for both electrodes. When it is small than the thicknesses of the electrodes, the heat generated in them can be considered as resulting from surface sources. Normally, the distance between the electrodes *d* is smaller than $l_0$, and the cathode and anode thicknesses $l_1,\,l_2$ are larger than $l_0$.

The cathode is metallic, with a significantly reduced work function. The anode is also metallic. Both electrodes have unequal Fermi energies. Quantum carbon heterostructures are more promising for low-threshold field emission^23^. They can be fabricated by vacuum magnetron sputtering from a low-pressure plasma of alternating layers (phases) of nanodiamond and nanographite clusters. The increased field emission from them is due to deep field penetration, large porosity and large emission surface area, and field weakening in the dielectric diamond phase, which reduces the barrier width^23^.

It is convenient to use n-layer graphene structures as grids. With four or five layers, a grid electrode more than 1 nm wide can be obtained, which is quite acceptable. Dispersion in the grids is not taken into account. The use of cathodes with a low emission threshold is not important for RT because for such structures the emission is mainly determined by the profile of a potential distribution. It is convenient to solve the SE by taking the origin of the quantum potential at the bottom of the double well. Then the potential in the well is zero, and only the potentials of the three barriers $V_n$, $n = 1,2,3$ have to be specified. The dimensions of the wells are comparable to the dimensions of the grids. Since we fix the distance *d*, we have to allocate the sizes of two barriers, which involves specifying the value of five parameters. These parameters will be assigned on the condition that the tunnel current of a complex profile coincides with the current of the considered structure.

We will analyse the energy balance in the steady state that the system reaches in which the temperature varies linearly from the electrodes to the thermostat and does not depend on time. The temperature gradient at the cathode $(T_1-T_0)/l_1$ induces a heat flow into the thermostat $k_1(T_1-T_0)/l_1$. The temperature gradient at the anode also creates a heat flow into the thermostat $k_2(T_2-T_0)/l_2$. Radiative heat flux between the cathode and the anode $S(T_1,T_2)$, which is a nonlinear function, must also be taken into account in the energy balance. One can assume that the temperature of a photonic field in the vacuum gap *T* is $(T_1+T_2)/2$. The total current is $J=S_0j$, with $S_0$ the area of the electrodes fulfilling the condition $d\ll \sqrt{S_0}$ for which tunneling is practically one-dimensional. Calling $\rho _1$ and $\rho _2$ the cathode and anode resistivities (Ohms/m), the heat transfer per unit surface from the cathode is $j^2\rho _1l_1$ or equivalently $R_1J^2/S_0$, where the cathode resistance is $R_1 =\rho _1 l_1/S_0$.

Suppose that all the electrons with energy *E* in the band $\Delta E$ undergo resonant tunnelling. The number of such electrons is

$$\begin{aligned} n_1=\frac{m_e}{2\pi ^2\hbar ^3}\Delta E(E_{F_1}-E)dE. \end{aligned}$$

The remaining electrons are reflected. Due to the Nottingham effect, the heat energy released along a free path reads

$$\begin{aligned} (E_{F_1}-E)n_1=(E_{F_1}-E)^2\frac{m_e}{2\pi ^2\hbar ^3}\Delta E. \end{aligned}$$

Here and hereafter we will assume that $\Delta E<<E$. Electrons falling on the anode acquire the energy $V_a=eU_a$ which contributes to increasing their mean free path. When this length is much smaller than the thickness of the electrodes, one can assume that the heat sources are superficial which implies that heat transfer is radiative, with *S* the heat flux. The reverse radiative heat flux can be ignored when the emission at the cathode is an order of magnitude greater than that at the anode. Assuming that the resonant tunneling occurs through one level, the energy balances at zero temperature in both electrodes are

17 $$\begin{aligned} &  (E_{F_1}-E_1)^2\frac{m_e}{2\pi ^2\hbar ^3}\Delta E_1+ \rho _1l_1\left[ \frac{em_eE_{F_1}\Delta E_1}{2\pi ^2\hbar ^3}\left( 1-\frac{E_1}{E_{F_1}}\right) \right] ^2=S+k_1(T_1-T_0)/l_1 \end{aligned}$$

18 $$\begin{aligned} &  (E-E_{F_2}+eV_a)(E_{F_1}-E_1)\frac{m_e}{2\pi ^2\hbar ^3}\Delta E_1+ \rho _2l_2\left[ \frac{em_eE_{F_1}\Delta E_1}{2\pi ^2\hbar ^3}\left( 1-\frac{E_1}{E_{F_1}}\right) \right] ^2=-S+k_2(T_2-T_0)/l_2. \end{aligned}$$

In these equations, the first term on the left-hand side account for the heating due to the transition of electrons from the cathode Fermi level to the anode Fermi level, and the second term is a consequence of Joule heating. The terms on the right-hand side come from the heat flows by conduction and radiation. The current density is thus

19 $$\begin{aligned} j=\frac{em_eE_{F_c}\Delta E_1}{2\pi ^2\hbar ^3}\left( 1-\frac{E_1}{E_{F_c}}\right) =\frac{em_eE_{F_1}\Delta E_1}{2\pi ^2\hbar ^3}(E_{F_c}-E_1). \end{aligned}$$

Taking into account 19 we obtain

20 $$\begin{aligned} &  (E_{F_1}-E_1)^2\frac{m_e}{2\pi ^2\hbar ^3}\Delta E_1+ j^2\rho _1l_1 =S+k_1(T_1-T_0)/l_1 \end{aligned}$$

21 $$\begin{aligned} &  (E-E_{F_2}+eV_a)(E_{F_1}-E_1)\frac{m_e}{2\pi ^2\hbar ^3}\Delta E_1+ j^2\rho _2l_2 =-S+k_2(T_2-T_0)/l_2. \end{aligned}$$

from which we obtain the electrode temperatures

22 $$\begin{aligned} &  T_1=T_0+(l_1/k_1)\left[ (E_{F_1}-E_1)^2\frac{m_e}{2\pi ^2\hbar ^3}\Delta E_1+j^2\rho _1l_1-S(T_1,T_2)\right] , \end{aligned}$$

23 $$\begin{aligned} &  T_2=T_0+(l_2/k_2)\left[ E_1-E_{F_1}+eU_a)j/e+j^2\rho _2l_2+S(T_1,T_2)\right] . \end{aligned}$$

In the case of several resonances, the temperatures are

24 $$\begin{aligned} &  T_1=T_0+(l_1/k_1)\left[ \sum _{n=1}^N\,D_n(E_{F_1}-E_1)^2\frac{m_e}{2\pi ^2\hbar ^3}\Delta E_1+j^2\rho _1l_1-S(T_1,T_2)\right] , \end{aligned}$$

25 $$\begin{aligned} &  T_2=T_0+(l_2/k_2)\left[ \sum _{n=1}^N\,D_nE_1-E_{F_1}+eU_a)j/e+j^2\rho _2l_2+S(T_1,T_2)\right] . \end{aligned}$$

As will be shown in the next section, the value $S(T_1,T_2)$ is small and can be ignored.

**Figure 5:**
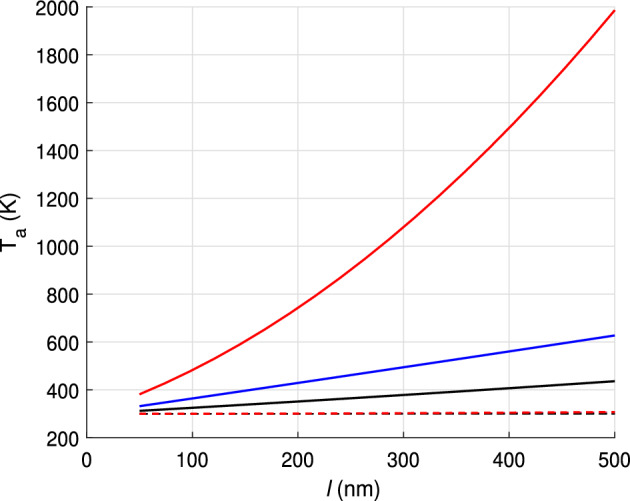
Cathode and anode temperatures versus the lengths of electrodes $l=l_1=l_2$. Blue and black solid lines correspond to the temperatures at the cathode in the 4-th and 1-st structures. The red line shows the temperature at the cathode made of diamond graphite with parameters of structure 4. The dashed red line shows the temperature at the anode in this structure. Temperatures at electrodes in other structures slightly differ from the thermostat temperature $T_0=300$ K.

We will first consider the case in which the anode and cathode are made of copper. Figure 5 illustrates how the temperatures $T_1$ and $T_2$ depend on the thicknesses of the cathode $l_1$ and anode $l_2$ which are taken to be equal. The thermal conductivity coefficient of copper is $k_1=400$ W/(m K) and the resistivity is $\rho _1=1.75\times 10^{-6}~\Omega$ m. The results show that if the electrode thicknesses do not exceed 500 nm their temperatures are close to that of the thermostat. The linear dependence of the temperature on the electrode thicknesses (see the blue and black lines) shows that the term with $j^2$ is negligibly small, which means that heating due to the Nottingham effect dominates over Joule heating.

In the second case analysed, the cathode material was diamond-graphite while the anode material was copper. The coefficient of thermal conductivity can be estimated from those of the two phases that make up the material. For graphite, the thermal conductivity coefficient varies from 100 to 354 W/(m K). For diamond, it is about 150 W/(m K), and for polycrystalline graphite in the form of nanoclusters, it is about 200 W/(m K), considering porosity. Taking into account that both phases have the same thickness, the value of the coefficient is about $k_1=175$ W/(m K). Regarding the resistivity, we will assume that $\rho _1=1.0\times 10^{-4}$, assuming that percolation theory is applicable to the heterostructure, or that tunnelling through low conductivity layers is possible. The red curve of Fig. 5 shows the increase of the cathode temperature with the thickness of the electrodes. The temperature dependence on $l_1$ is in this case non-linear, which implies that Joule heating is in this case relevant.

## Poynting vector and radiative heat transfer

Near-field heat transfer between two closely spaced media can exceed by orders of magnitude the intensity of radiation emitted by a black body due to the photon tunneling effect caused by evanescent waves. Since the distance between the anode and cathode is only a few nanometres, we have to estimate the contribution of near-field radiative heat transfer to the total energy balance. In our analysis, we will follow Ref.^35^ by applying the transfer matrix method to take into account the presence of grids in the gap^36^.

The spectral density of the total heat transferred by electromagnetic waves between the two media at different temperatures is (see Fig. 1)^35^

26 $$\begin{aligned} S_{\omega }=\int _0^{\infty }\,\left[ \langle S_z^{12}(k_x,\omega ,T_1)\rangle -\langle S_z^{21}(k_x,\omega ,T_2)\rangle \right] k_xdk_x. \end{aligned}$$

where we have neglected radiation and heat absorption in the grids. Since we consider a stationary regime, the radiation absorbed by the carbon nanostructures is irradiated again. This approach is justified in thin layers of graphene or CNT. We will consider the effect of the grids through the transmission coefficient expressed in terms of the elements of the transfer matrix.

To calculate the Poynting vectors $\langle S_z^{12}\rangle$ and $\langle S_z^{21}\rangle$ , we first find the incident Poynting vector $\langle S_z^{1}(k_x,\omega )\rangle$ calculated for the semi-infinite medium 1 neglecting the reflection from the interface between the medium 1 and the gap between media 1 and 2. Then we apply the transfer matrix method, which allows us to express $\langle S_z^{12}\rangle$ through the incident Poynting vector $\langle S_z^{1}(k_x,\omega )\rangle$ and the values of parameters of the medium placed in the gap. Following Ref.^35^, we start from Maxwell’s inhomogeneous equations with a random electric current density source *j* and apply the fluctuation-dissipation theorem^37,38^ for the ensemble-averaged bulk current density:

27 $$\begin{aligned} \langle j_m(\omega ,k_x)j_n(\omega ',k_x')\rangle =\frac{1}{\pi }\omega \epsilon _0\ E_m''\delta _{mn}\delta (\omega -\omega ')\delta (k_x-k_x')\Theta (\omega ,T), \end{aligned}$$

where $\delta _{mn}$ is the Kronecker symbol, $\delta (x)$ is the Dirac $\delta$- function,

28 $$\begin{aligned} \Theta (\omega ,T)=\frac{\hbar \omega }{\exp {\left( \frac{\hbar \omega }{k_BT}-1\right) }}, \end{aligned}$$

is Planck’s distribution, $\epsilon _m''$ is the imaginary part of the relative permittivity of medium *m*, ($m=1,2$), and $k_B$ is Boltzmann’s constant.

Due to the homogeneity of media 1 and 2 in the (*x*, *y*) plane, a bulk current source in the form of a harmonic current sheet may be used:

29 $$\begin{aligned} j(z) = j_0(z')\delta (z-z')e^{-i(\omega t- k_xx - k_yy)}, \end{aligned}$$

Then we calculate the ensemble-averaged $\langle E_xH_y\rangle$ using the fluctuation-dissipation theorem to finally obtain the result^35,36^:

30 $$\begin{aligned} \langle S_z^1(k_x,\omega )\rangle =\frac{\epsilon _1''(\omega )}{2\pi \epsilon _1k_{1z}\text{Im}(k_{1z})}(k_x^2+k_{1z}k_{1z}^*)\Theta (\omega ,T_1)+\mathrm{c.c} \end{aligned}$$

where $k_{(1,2)z}=\sqrt{k_0^2\epsilon _{1,2}-k_x^2-k_y^2}$, $k_0$ is the wavenumber in vacuum, and c.c. denotes complex conjugate.

Now we have to obtain the Poynting vector transmitted from medium 1 to medium 2 taking into account the reflections of the waves at the cathode-anode interface. Assuming that Maxwell’s boundary conditions are satisfied at both boundaries, we express the Poynting vector through the waves transmission coefficient ${\mathcal {T}}$ and the transverse wave impedances of the media:

31 $$\begin{aligned} \langle S_z^{12}(k_x,\omega )\rangle =\frac{1}{2}\langle S_z^1(k_x,\omega )\rangle |{\mathcal {T}}|^2\frac{Z_1^*}{Z_2^*} + \mathrm{c.c.}, \end{aligned}$$

where $$Z_i=-E_x/H_y=\eta k_{iz}/(k_0\epsilon _i)$$ ($$i=1,2)$$, and $$\eta =120\pi \,\Omega $$ is the wave impedance of vacuum. The coefficient $${\mathcal {T}}$$ can be obtained through the transfer matrix components of the gap between media 1 and 2:

32 $$\begin{aligned} {\mathcal {T}}=\frac{2}{B_{11}+B_{12}/Z_2+B_{21}Z_1+B_{22}Z_1/Z_2}, \end{aligned}$$

where $B_{mn}, \;m,n=1,2$ are elements of the electromagnetic transfer matrix ${\mathbb {B}}$ which is the product of five transfer matrices corresponding to three segments of vacuum gaps and two grids: ${{\mathbb {B}}}=\tilde{{\mathbb {A}}^1} \tilde{{\mathbb {A}}^g} \tilde{{\mathbb {A}}^2} \tilde{{\mathbb {A}}^g} \tilde{ {\mathbb {A}}^3}$, similarly to the electron tunneling. The transfer matrices $\tilde{{\mathbb {A}}^n}, n=(1,2,3)$ have elements

33 $$\begin{aligned} \begin{array}{ccc} {\tilde{a}}^n_{11}={\tilde{a}}^n_{22}=\cos {(k_{0z}t_n)},&\tilde{a}^n_{12}=iZ_0\sin {(k_{0z}t_n},&\tilde{a}^n_{21}=i/Z_0\sin {(k_{0z}t_n)}, \end{array} \end{aligned}$$

where $$k_{0z}=\sqrt{k_0^2-k_x^2-k_y^2}$$.

In turn, the transfer matrix of a grid $\tilde{{\mathbb {A}}^g}$ is the product of transfer matrices corresponding to graphene interlayer vacuum gaps $d_g$ contacted with graphene sheets, so $\tilde{\mathbb A^g}={{\mathbb {M}}}^N$ where *N* is the number of graphene sheets. The elements of the matrix ${{\mathbb {M}}}$ read as

34 $$\begin{aligned} \begin{array}{lr} M_{11}=\cos {(k_{0z}d_g)}, & M_{12}=iZ_0\sin {(k_{0z}d_g)}, \\ M_{21}=i/Z_0\sin {(k_{0z}d_g)} +\sigma \cos {(k_{0z}d_g)} & M_{22}= \cos {(k_{0z}d_g)} +i\sigma Z_0\sin {(k_{0z}d_g)}, \end{array} \end{aligned}$$

where $\sigma$ is the surface conductivity of graphene given by Kubo’s formula^39^, and $d_g$ is taken to be 0.335 nm.

**Figure 6:**
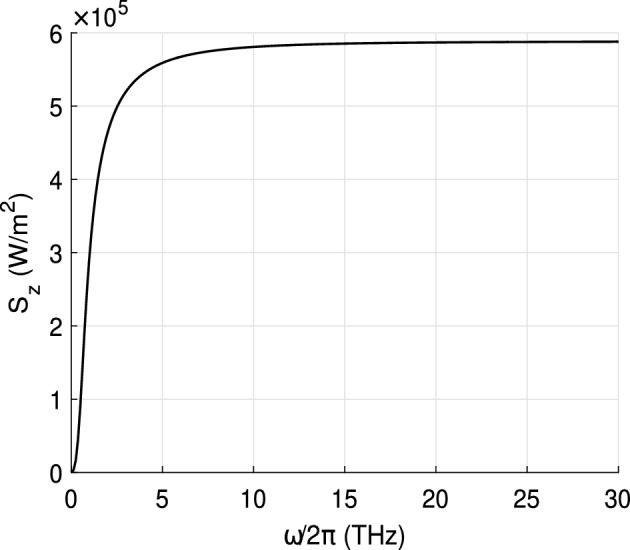
Radiative heat transfer per unit square between the cathode and anode. The distance between the cathode and anode *d*=10 nm. Temperatures of the cathode and anode are 1500 K and 900 K, respectively.

Figure 6 illustrates the result of the numerical integration over frequency of the difference between the Poynting vectors

$$\begin{aligned} S_z=\int _0^{\infty }S_{\omega }\,d\omega \end{aligned}$$

emitted by the cathode at $T_c$=1500 K and the anode at $T_a$=900 K. The integration stops when the integral converges which allows us to identify the frequency range contributing to the total thermal energy flow. Calculations show that the influence of the grids can be neglected. Thus, we obtain a radiative heat transfer from the cathode to the anode of about $\approx 6\times 10^5$  W/m$^2$ which is much smaller than conductive heat transfer of about $\approx 4\times 10^{12}$  W/m$^2$.

## Conclusions

Carbon field emission structures are known to have low emission thresholds and high electron emissivity. High emission currents can be achieved due to the resonant tunneling in vacuum heterostructures with potential barriers. Such high current densities generate a significant amount of heat, resulting in a considerable temperature increase of the structure to the point of damage. It is therefore important to be able to control the thermal effects on such structures. This has been the objective of the article.

Carbon structures with low thresholds are usually porous, which can make it challenging to dissipate heat. As a result, it would be best if the electrodes are constructed from a metal that has excellent thermal conductivity and a low work function. Using cryogenic cooling of the thermostats could be a potential approach to decrease operating temperatures. It would be possible to measure electrode temperatures and currents in existing semiconductor resonant tunnel diodes and transistors based on AlAs and AlGaAs technology. Our results could also be verified in vacuum resonant tunnel structures. In our study, we have also taken into account the contribution of radiative heat transfer to the heat balance. We have found that, for the cases studied, it is negligible.

We have analysed the stationary thermal regimes of triode-resonant-tunnel structures by calculating the temperature as a function of current density. We have shown that, depending on the nature of the electrode materials and their thicknesses, the temperature can be very similar to that of the bath or increase considerably. This result would make it possible to create structures with the desired current densities without generating excessive heat. Small values of $l_1$ and $l_2$ and good thermal conductivities result in good temperature control. In our study, we have also taken into account the contribution of radiative heat transfer to the heat balance. We have found that, for the cases studied, it is negligible.

We have shown that the quantum potential in Fig. 2, which requires a few hundred partitions for an accurate description, can be approximated with good accuracy by a profile with three rectangular barriers and two quantum wells. For a given profile, it is sufficient to find metastable energy levels to describe the current in the structure. To calculate the current, it is possible to replace the integral in its expression with the sum over the specified energy levels, since outside the resonant peaks the electron energy decreases exponentially, see Fig. 4. Therefore, the contribution of the tunnelled electrons outside the transmission peaks is very small and can be ignored.

The study of the resonant electron tunneling current and the resulting thermal effects we have presented allows us to identify optimal conditions for the correct operation of field-emitting triode structures useful in many applications.

## Data Availability

The data that support the findings of this study are available from the corresponding authors upon reasonable request.
